# Complete chloroplast genome of *Mesua ferrea*: the first Calophyllaceae plastome

**DOI:** 10.1080/23802359.2019.1666668

**Published:** 2019-09-17

**Authors:** Yi Wang, Xiaolong Yuan, Yunqing Li, Jinfeng Zhang

**Affiliations:** Laboratory of Forest Plant Cultivation and Utilization, Yunnan Academy of Forestry, Kunming, People’s Republic of China

**Keywords:** *Mesua ferrea*, chloroplast, Illumina sequencing, phylogenetic analysis

## Abstract

The first complete chloroplast genome sequences of *Mesua ferrea* were reported in this study. The cpDNA of *M. ferrea* is 161,470 bp in length, contains a large single copy region (LSC) of 88,760 bp and a small single copy region (SSC) of 17,482 bp, which were separated by a pair of inverted repeat (IR) regions of 27,614 bp. The genome contains 131 genes, including 86 protein-coding genes, eight ribosomal RNA genes, and 37 transfer RNA genes. The overall GC content of the whole genome is 36.4%. Phylogenetic analysis of 30 chloroplast genomes within the order Malpighiales suggests that *M. ferrea* is closely related to *Garcinia mangostana*.

*Mesua ferrea* belongs to the genus *Mesua* in Calophyllaceae (Calophylleae) is distributed in Asia’s tropical countries like India, Burma, Thailand, China, and New Guinea (Rajalakshmi et al. 2019). It is one of the famous hardwoods in tropical Asia. Introduction and cultivation of *M. ferrea* have a history of 500 years in China (Ming et al. 2015). The extracts of *M. ferrea* showed anticancer bioactive on human pancreatic cancer cell line (Rajendran et al. 2016). Recently, researchers confirmed that the anticancer bioactive compound of *M. ferrea* was terpene (Asif et al. 2017). The extract of *M. ferrea* also showed antiarthritic, antioxidant, and antibacterial activity (Jalalpure et al. 2011; Chahar et al. 2013). Therefore, *M. ferrea* has huge potential medicinal value. However, there has been no genomic studies on *M. ferrea*.

Herein, we reported and characterized the complete *M. ferrea* plastid genome (MN052680). One *M. ferrea* individual (specimen number: 201803001) was collected from Jinghong, Yunnan Province of China (22°49′52″ N, 101°9′89″ E). The specimen is stored at Yunnan Academy of Forestry Herbarium, Kunming, China, and the accession number is YAFH0012718. DNA was extracted from its fresh leaves using DNA Plantzol Reagent (Invitrogen, Carlsbad, CA, USA).

Paired-end reads were sequenced using Illumina HiSeq system (Illumina, San Diego, CA). In total, about 23.1 million high-quality clean reads were generated with adaptors trimmed. Aligning, assembly, and annotation were conducted by CLC de novo assembler (CLC Bio, Aarhus, Denmark), BLAST, GeSeq (Tillich et al. 2017), and GENEIOUS v 11.0.5 (Biomatters Ltd, Auckland, New Zealand). To confirm the phylogenetic position of *M. ferrea*, other 29 species of Order Malpighiales from NCBI were aligned using MAFFT v.7 (Katoh and Standley 2013) and maximum-likelihood (ML) bootstrap analysis was conducted using RAxML (Stamatakis 2006); bootstrap probability values were calculated from 1000 replicates. *Quercus baronii* (KT963087) and *Quercus dentata* (MG967555) were served as the out-group.

The complete *M. ferrea* plastid genome is a circular DNA molecule with the length of 161,470 bp, with large single copy (LSC: 88,760 bp), small single copy (SSC: 17,482 bp), and two inverted repeats (IRa and IRb: 27,614 bp each). The overall GC content of the whole genome is 36.4%, and the corresponding values of the LSC, SSC, and IR regions are 34.0%, 30.6%, and 42.1%, respectively. The genome contains 131 genes, including 86 protein-coding genes, eight ribosomal RNA genes, and 37 transfer RNA genes. Phylogenetic analysis showed that *M. ferrea* clustered together with *Garcinia mangostana*, which indicated the phylogenesis classification of *M. ferrea* (Figure 1). The determination of the complete plastid genome sequences provided new molecular data to illuminate the Malpighiales evolution.

**Figure 1.**
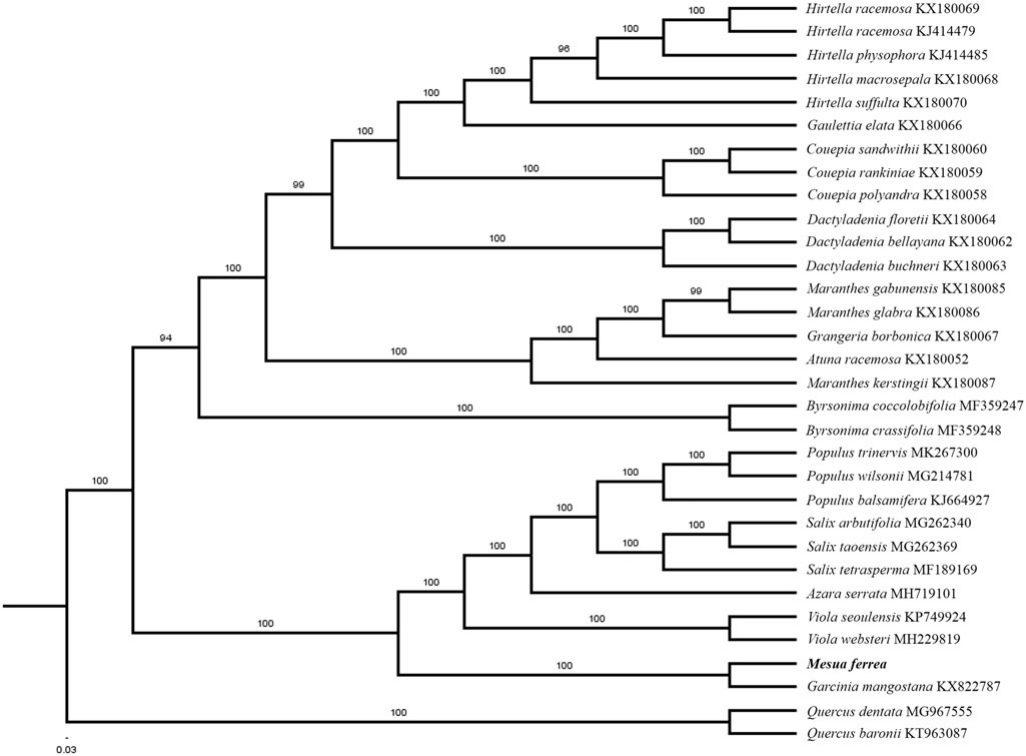
The maximum-likelihood tree based on the 30 chloroplast genomes of order Malpighiales. The bootstrap value based on 1000 replicates is shown on each node.
